# Effectiveness of One-Stop Screening for Colorectal, Breast, and Prostate Cancers: A Population-Based Feasibility Study

**DOI:** 10.3389/fonc.2021.631666

**Published:** 2021-02-25

**Authors:** Joseph J. Y. Sung, Arthur K. C. Luk, Simon S. M. Ng, Anthony C. F. Ng, Peter K. F. Chiu, Emily Y. Y. Chan, Polly S. Y. Cheung, Winnie C. W. Chu, Sunny H. Wong, Thomas Y. T. Lam, Samuel Y. S. Wong

**Affiliations:** ^1^ Institute of Digestive Disease, The Chinese University of Hong Kong, Hong Kong, Hong Kong; ^2^ Department of Medicine and Therapeutics, The Chinese University of Hong Kong, Hong Kong, Hong Kong; ^3^ Department of Surgery, The Chinese University of Hong Kong, Hong Kong, Hong Kong; ^4^ Jockey Club School of Public Health and Primary Care, The Chinese University of Hong Kong, Hong Kong, Hong Kong; ^5^ Hong Kong Breast Cancer Foundation, Hong Kong, Hong Kong; ^6^ Department of Imaging and Interventional Radiology, The Chinese University of Hong Kong, Hong Kong, Hong Kong

**Keywords:** cancer screening, colorectal cancer, breast cancer, prostate cancer, one-stop approach

## Abstract

Colorectal cancer (CRC), prostate cancer (PC) and breast cancer (BC) are among the most common cancers worldwide with well-established screening strategies. We aim to investigate the effectiveness and compliance of a one-stop screening service for CRC, PC and BC. Asymptomatic subjects aged 50–75 years were invited. Eligible subjects were offered fecal immunochemical test (FIT) for CRC screening. Serum prostate specific antigen (PSA) and Prostate Health Index (PHI) were offered for male PC screening and mammogram (MMG) for female BC screening as a one-stop service. Colonoscopy was offered to FIT+ subjects, prostate biopsy to PSA/PHI+ (PSA>10/PHI≥35) males and breast biopsy to MMG+ (Breast Imaging-Reporting and Data System, BI-RADS≥4) females. From August 2018 to April 2020, 3165 subjects were recruited. All participants (1372 men and 1793 women) were willing to accept FIT for CRC screening, and PSA/PHI test or MMG as second cancer screening. 102 subjects diagnosed advanced neoplasms after colonoscopy. Thirty-three males diagnosed PC after prostate biopsy and 15 females diagnosed BC after breast biopsy. No major complication reported in first tier screening tests. Subjects who were willing to undergo CRC screening were highly likely to accept other cancer screening when offered in a one-stop program. In conclusion, the effectiveness and compliance of a one-stop service for CRC, PC, and BC screening among asymptomatic subjects were high. Future studies should be conducted to test various ways of integrating cancer screening programs.

**Clinical Trial Registration:**

ClinicalTrials.gov, identifier NCT04034953.

## Introduction

Cancer represents a major global health burden, and dying from cancer is now more common than death from cardiovascular disease especially in high income countries (1). The GLOBOCAN 2018 statistics has estimated that across twenty world regions, there was 18.1 million new cancer cases and 9.6 million cancer deaths in 2018 (2). Lung cancer is the most frequent cancer among men, followed by prostate and colorectal cancer for incidence. Among females, breast cancer is the most commonly diagnosed cancer and the leading cause of cancer death.

Among these most common cancers, screening and early diagnosis offer the best chance of cure and survival. While lung cancer screening for current or former smokers with low-dose helical CT scan is adopted in the US, the recommendation is still under discussion in Europe and other parts of the world (3, 4). On the other hand, breast cancer screening of women age 45–54 years by annual or biennial mammogram, colorectal cancer screening by fecal immunochemical test or colonoscopy from the age of 50 years, and prostate cancer screening by prostate-specific antigen test with or without digital rectal examination from the age of 50 years are widely accepted guidelines proposed by national and international guidelines (5–11).

While clinical trials have proven the effectiveness of screening for colorectal cancer, prostate cancer and breast cancer, uptake of the screening tests by asymptomatic individuals is a major hurdle to the success of screening program worldwide. In the US, screening rate for breast cancer is 32.1%, cervical cancer 36.1% and colorectal cancer 30.1% (12). Less than 10% of women had completed screening for all three screening tests according to guidelines. Cancer screening guideline for breast, cervical and colorectal cancers were followed by women over 50 years of age in Canada in only 8% to 43% (13). The screening adherence across breast, cervical and colorectal cancers are rare.

Unlike screening for chronic diseases such as diabetes, hypertension and hyperlipidemia, cancer screening is often launched for a single disease. It is worth considering whether these preventive services can be integrated into a multiple screening program that includes the most common cancers and their related pre-cancerous conditions. The advantages of a multiple screening program, over individualized screening for each disease, include the provision of one-stop service that may improve efficacy and uptake rate, and reduction of duplicated manpower and logistics. Integrated cancer screening for women over 50 years including breast, cervical and colorectal cancers has been tested. In a systematic review of seven studies, overall adherence was only 8% to 43%, and reports of screening adherence across breast, cervical, and colorectal cancers are rare (13). In an Israeli study, screening tests for prevention and early detection of 11 cancers were offered to asymptomatic individuals (14, 15). The screening program included not only routine tests such as mammography, colonoscopy, PSA, but also consultation by experts and annual examination. While the concept of multiple cancer screening is attractive, the workload and complexity have rendered this approach non-sustainable as a population-wide public health policy. In Taiwan, a community-based multiple screening model including tests for five cancers: breast, liver, colon/rectum, cervix and oral cavity has been evaluated. There is also inclusion of testing for diabetes, hypertension and hyperlipidemia (16). The integrated screening program has claimed to increase the attendance rate of Pap smear by 25% and found asymptomatic neoplasms in 16% of the asymptomatic population. However, a multi-dimensional screening program requires a sophisticated follow up program to findings from screening test, such as mammography, fecal occult blood test, Pap smear, ultrasonography and oral examination. The program was ambitious, and it has not been taken up by the public health system.

We now describe our new model of multiple cancer screening program and present the pilot study results. This cancer screening program includes screening for colorectal cancers, and the addition of prostate cancer screening for men and breast cancer screening for women. We aimed to investigate the effectiveness and compliance of a one-stop service for colorectal, prostate and breast cancers screening among asymptomatic subjects.

## Materials and Methods

This is a community-based project sponsored by a charitable organization, the Hong Kong Jockey Club Charities Trust, under the auspice of the Faculty of Medicine at the Chinese University of Hong Kong. The Multiple Cancer Screening Center (MCSC) enrolled subjects for screening through media promotion and advertisement, including the use of conventional media (newspaper, radio and television). Subjects who were interested to be included for cancer screening were registered online, by telephone or walk-in. They were then contacted by trained personnel by phone to confirm eligibility. They were first briefed about the eligibility of colorectal cancer (CRC) screening (see below). During the phone interview, they were also informed that there were options to join the prostate cancer (PC) screening for male or breast cancer (BC) screening for female. Subjects who accepted CRC screening were invited to visit our community center on a scheduled appointment for further explanation of the CRC program as well as options for BC and PC screening.

Asymptomatic male and female subjects over the age of 50 years were recruited for CRC screening. Exclusion criteria included personal history of CRC or colectomy; hematochezia, melena, anorexia or change in bowel habit in the past 4 weeks, or weight loss of greater than 5 kg in the past 6 months; having received FIT in the past 2 year; flexible sigmoidoscopy in the past 5 years; colonoscopy in the past 10 years; strong family history of CRC (two or more first degree relative diagnosed CRC), personal history of colonic adenoma, diverticular disease, inflammatory bowel disease, prosthetic heart valve or vascular graft surgery; medical conditions which were contraindications for colonoscopy. They were then offered a fecal immunochemical test (OC-Sensor, Eiken Chemical Co. Ltd., Tokyo, Japan) and asked to return the test within 8 weeks. Those who were tested positive were referred to the Prince of Wales Hospital Endoscopy Center for colonoscopy.

For male subjects, after accepting CRC screening, they were given an option for joining PC screening. A workshop was offered to the subject on the same day, during which knowledge about presentation and management of PC were provided. The potential benefits and risks of PC screening, i.e. blood for prostate specific antigen (PSA) and prostate health index (PHI) tests and prostate biopsy were introduced. If the male subject agreed to join PC screening, blood samples for PSA/PHI were taken on site. Ultrasound-guided prostate biopsy for PSA/PHI positive subjects (PSA > 10 ng/ml or PHI ≥ 35, for PSA level between 4–10 ng/ml). Exclusion criteria for PC screening include hematuria; personal history of prostate cancer; significant medical conditions that may result in limited life expectancy (< 10 years) of the screening participant; and having received any PC screening test in the past 5 years.

For female subjects, after accepting CRC screening, they were given an option for joining BC screening. A workshop was offered to the subject on the same day, during which knowledge about presentation and management of BC were provided. The potential benefits and risks of BC screening, i.e. mammography and breast lump biopsy were introduced. If the female subject agreed to join BC screening, mammogram (MMG) would be arranged at the earliest possible appointment at the same site. Ultrasound-guided breast biopsy would be offered to MMG-positive subjects (those with Breast Imaging-Reporting and Data System [BI-RADS] category ≥4). Exclusion criteria for BC screening include personal history of breast cancer; swelling of all or part of the breast; skin irritation or dimpling; breast pain; nipple pain or the nipple turning inward; redness, scaliness or thickening of the nipple or breast skin; a nipple discharge other than breast milk; lump in the underarm area; and having received any BC screening test in the past 5 years.

**Figure 1:**
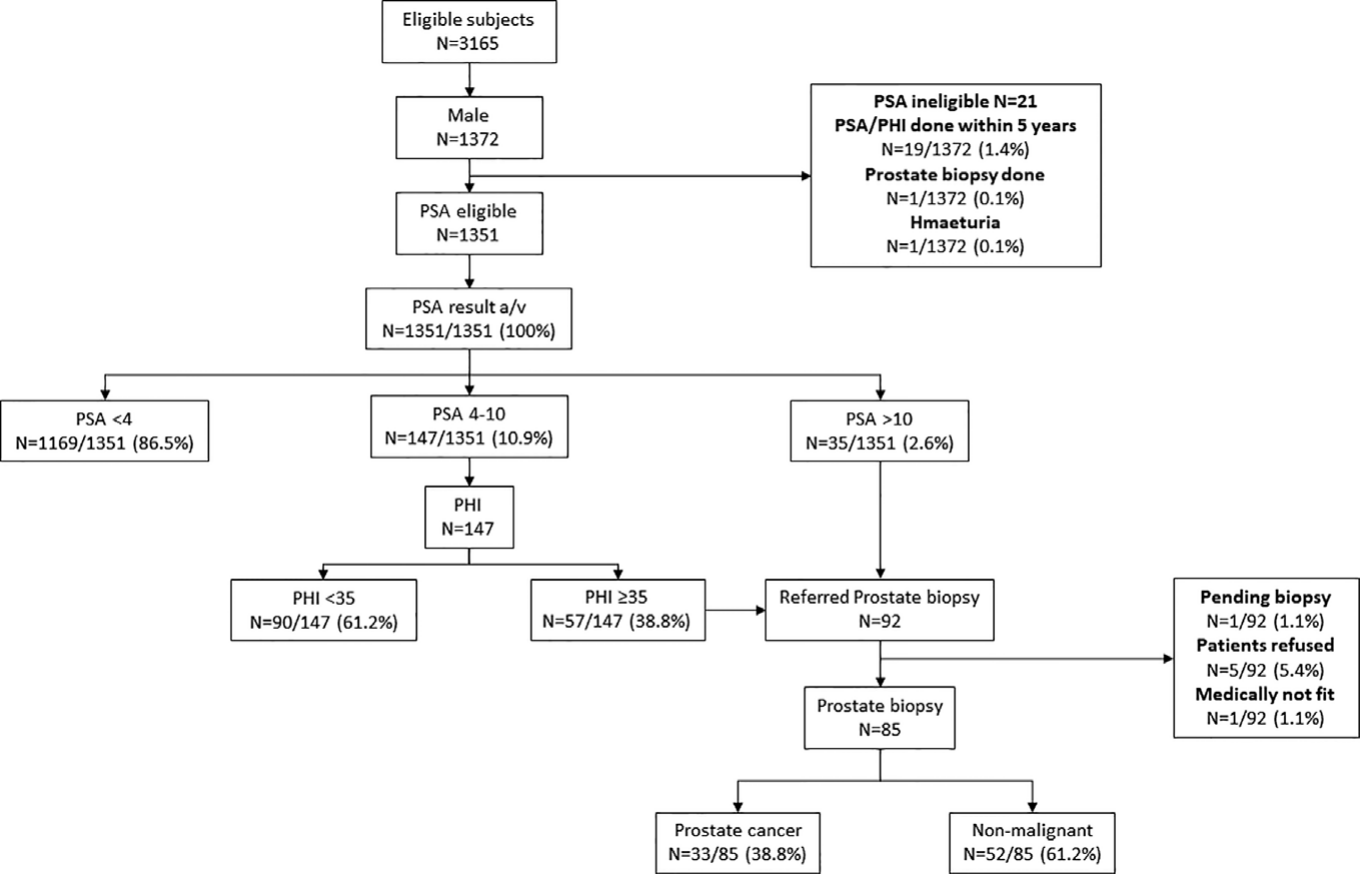
Study flow. CRC, colorectal cancer; PC, prostate cancer; BC, breast cancer; FIT, fecal immunochemical test; PSA, prostate surface antigen; PHI, prostate health index; and MMG, mammogram.

Subjects who refused to join PC or BC screening could still proceed with CRC screening alone. Briefing of CRC, PC, and BC screening were offered by trained nurses. Family physicians were present on site to give necessary counselling. All screening procedures were offered free of charge. When there was cancer diagnosed, precancerous lesion identified (e.g. colorectal neoplasia, breast lump), raised serum PSA with negative biopsy, patients would be referred to a hospital/clinic for further follow up and management with a letter from the MCSC. Subsequent management of the patients in private or public health services were paid by the subjects (**Figure 1**).

This study was approved by the Joint Chinese University of Hong Kong—New Territories East Cluster Clinical Research Ethics Committee (CRE-2018.165) and registered on clinicaltrials.gov (NCT04034953).

## Results

The screening program was started in Aug 2018; as of Apr 2020, a total of 3,165 subjects (mean age 62 ± 4.6) were recruited of whom 43.3% were male. 2,975 (94.0%) subjects were eligible for colorectal cancer screening, while 1,351 (42.7%) and 1,735 (54.8%) subjects were eligible for prostate and breast cancer screening, respectively. The mean age of this group was 62 years (SD 4.6 years) (**Table 1**). Only 53 (1.7%) subjects received no formal education, while 18.6%, 59.5%, 3.1%, and 17.1% received primary, secondary, vocational college and university education, respectively. Regarding family income (HKD per month), these subjects were quite evenly distributed in the five income groups (HKD ≤10,000: 18.1%; HKD10,001–20,000: 19.7%; HKD 20,001–30,000: 16.6%, HKD 30,001–40,000: 10.7%, >HKD 40,000: 12.9%; no information 22.1%).

**Table 1:** Demographic data of 3,165 asymptomatic subjects.

Total number of subjects	3165
Number of subjects eligible for colorectal cancer screening	2975
Number of subjects eligible for prostate cancer screening	1351
Number of subjects eligible for breast cancer screening	1735
Male Gender (%)	1372/3165 (43.3)
Mean age (SD)	62 (4.6)
BMI≥25 (%)	1877/3165 (59.3)
Diabetes (%)	615/3165 (19.4)
Hypertension (%)	1117/3165 (35.3)
Ischemic Heart Disease (%)	50/3165 (1.6)
Chronic Obstructive Pulmonary Disease (%)	44/3165 (1.4)
Stroke (%)	58/3165 (1.8)
Fatty Liver (%)	327/3165 (10.3)
Cirrhosis (%)	3/3165 (0.1)
Gastroesophageal Reflux Disease (%)	276/3165 (8.7)
Smoking (Current or Past) (%)	354/3165 (11.2)
Drinking (%)	324/3165 (10.2)
First degree relative colorectal cancer history (%)	372/2975 (12.5)
First degree relative prostate cancer history (%)	49/1351 (3.6)
First degree relative breast cancer history (%)	170/1735 (9.8)

### Eligibility and Recruitment

Since the program launch in 2018, 38,157 asymptomatic subjects have registered online showing interest for the program. To date, follow-up telephone contact of 11878 subjects (3 attempts to call for each registrant) have been made. 3,788 could not be reached by telephone, 3,415 were found to be ineligible for screening, 808 indicated they were no longer interested, 223 had the screening test done by other providers, and 311 indicated that they were busy and 168 declined for miscellaneous reasons. 3,165 subjects were confirmed to be eligible to join the screening program. All subjects were interested in CRC screening and one of the other screening (BC or PC) program.

### Colorectal Cancer Screening

**Figure 2:**
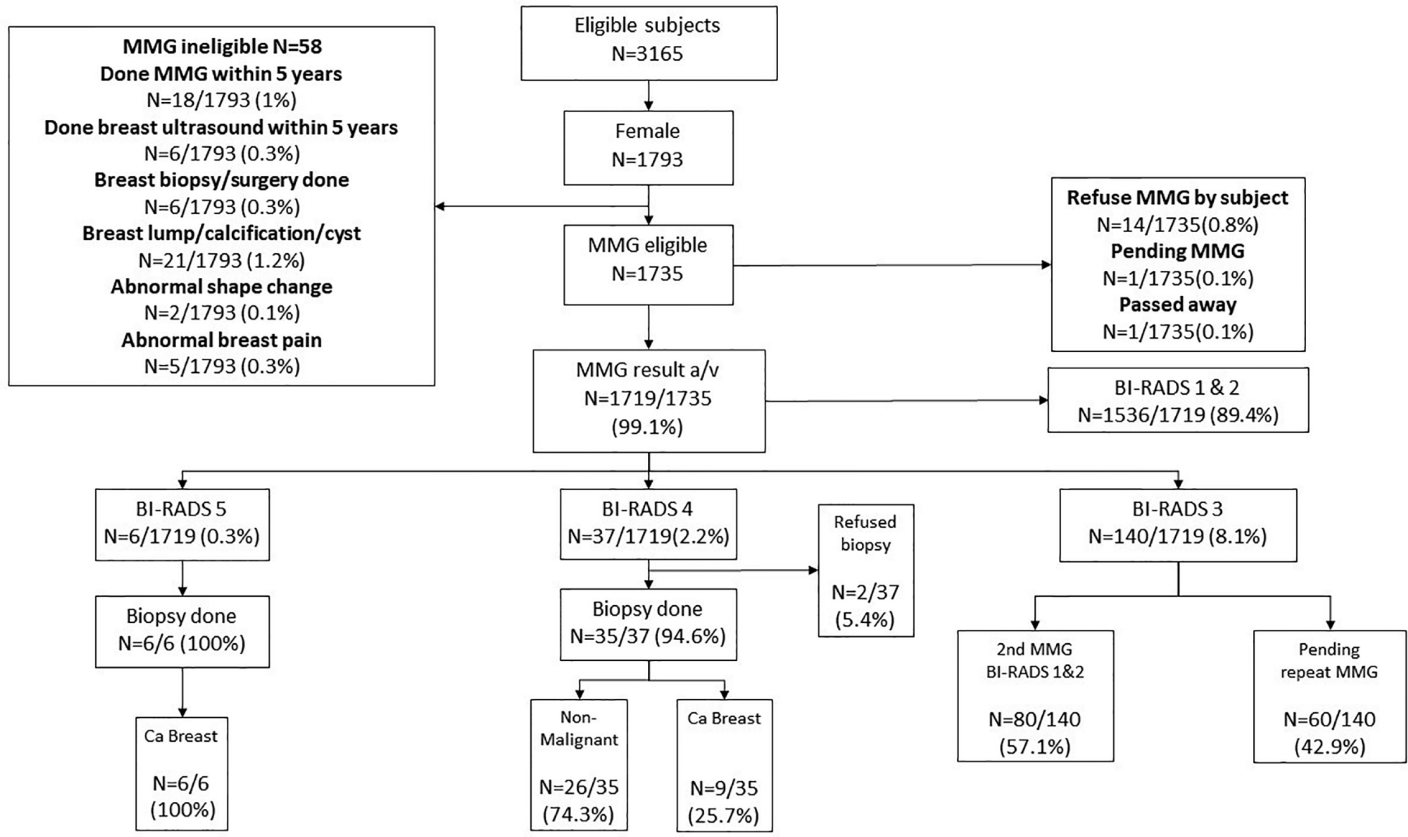
Colorectal cancer screening results. FIT—Fecal immunochemical test, a tool for colorectal cancer screening by identifying the fecal hemoglobin concentration. Our program provided two FITs for each subject at the same time. If either one of the tubes is tested positive, the subject will be referred to colonoscopy as follow up. For FIT results, if subjects misuse or did not return the kits within 8 weeks for the first time, they can have one chance to recollect the FIT kits and perform the test one more time. But if they failed for the second time, they will then need to retest 2 years later.

Three thousand one hundred sixty-five subjects were eligible for the CRC screening program, of whom only 2,975 were entitled for the FIT test. Two thousand nine hundred twenty-six subjects returned a valid FIT test, and among them 347 (11.9%) were tested positive. These patients were offered colonoscopy. One patient was considered medically unfit for colonoscopy, one is still pending colonoscopy at the time of writing of this manuscript, five turned down the offer, and one passed away before colonoscopy could be arranged. Among the 339 subjects who had completed colonoscopy examination, adenomas were found in 148 (43.7%), advanced adenoma in 93 (27.4%) and CRC in 9 (2.7%) (**Figure 2**). No complication relate to sedation or endoscopy was reported.

### Prostate Cancer Screening

**Figure 3:**
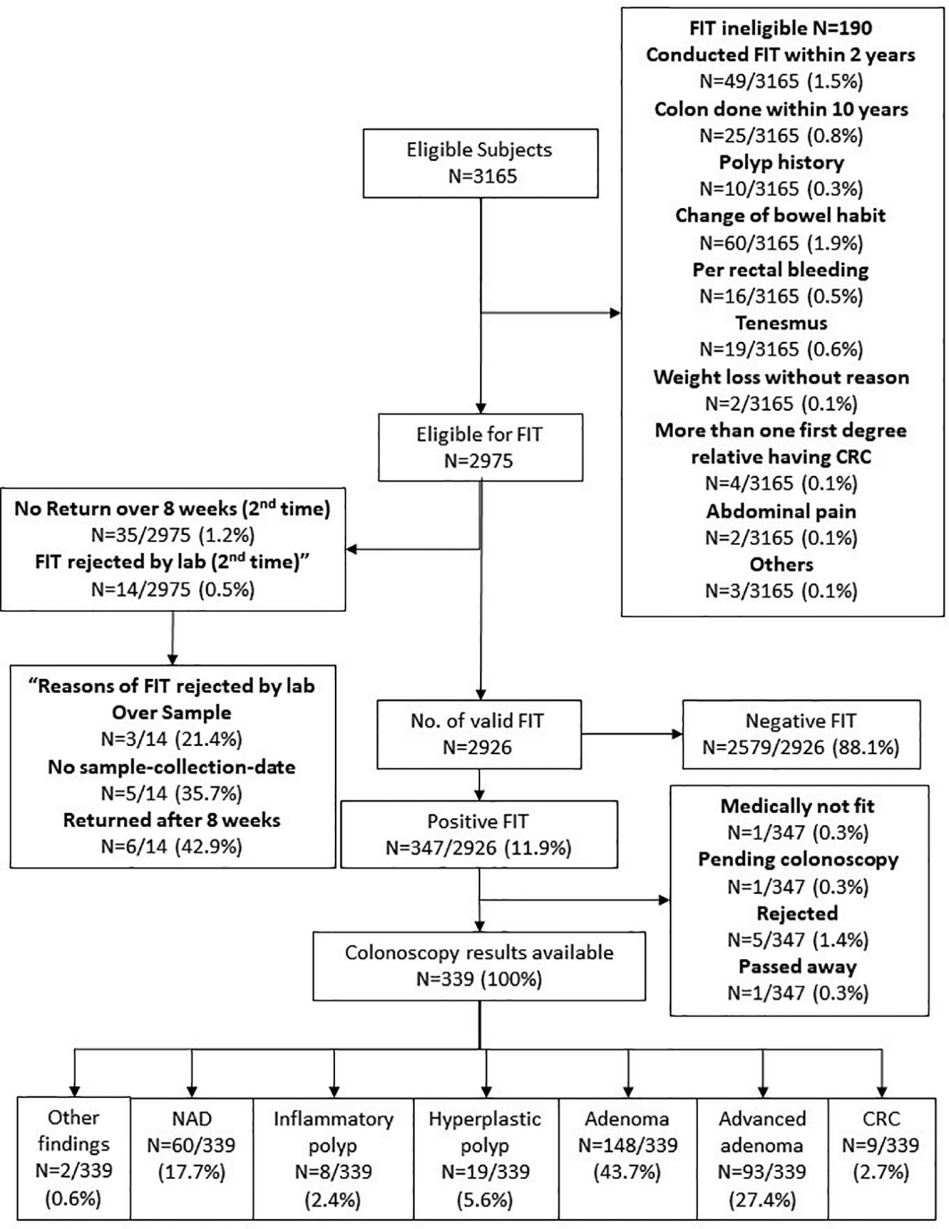
Prostate Cancer screening results. PSA, Prostate specific antigen; PHI, Prostate Health Index. PHI is a formula that combines total PSA, free PSA and p2PSA into one score for decision making on whether to conduct further follow up such as prostatic biopsy. Our program offered PSA for all male subjects. With PSA score 4–10, our laboratory will conduct PHI for the subject. If the PHI score is >35, the subject will be referred to urologist for consultation and prostatic biopsy, which is the same treatment if PSA >10.

One thousand three hundred fifty-one male subjects who were enrolled in the CRC screening program also accepted the invitation for PC screening by serum PSA. Thirty-five subjects had serum PSA >10 ng/ml were referred for prostate biopsy. Among the 147 subjects who had serum PSA 4–10 ng/ml, 57 had PHI ≥35, and they were also referred for prostate biopsy. Altogether, 92 subjects were referred for prostate biopsy. Among them, five refused the investigation, one was considered medically unfit for biopsy, and one is still awaiting biopsy. prostate biopsy at the time of writing of this manuscript. Among those who had a successful prostate biopsy (n = 85), 33 had confirmed prostate cancer (38.8%) (**Figure 3**). Three (3.5%) patients developed sepsis after prostate biopsy and recovered after antibiotic treatment.

### Breast Cancer Screening

**Figure 4:**
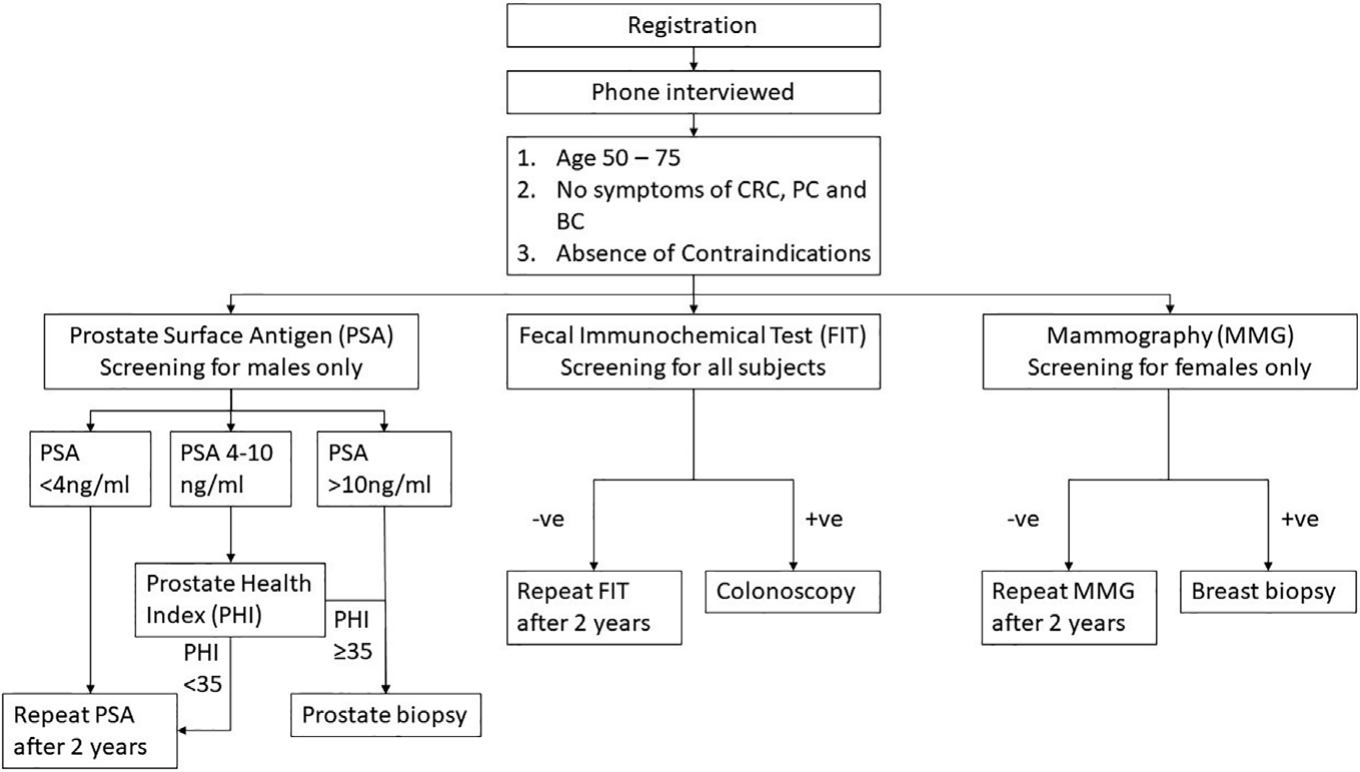
Breast Cancer screening results. MMG, Mammogram; BI-RADS, Breast Imaging Reporting and Data System. BI-RADS 1 and 2 subjects will be considered as negative and to repeat MMG in 2 years. For BI-RADS 3 subjects, another MMG will be arranged in 6–12 months. Subjects who were diagnosed with BI-RADS 4 and 5 will be considered as MMG positive and ultrasound-guided breast biopsy will be offered as follow up.

One thousand seven hundred thirty-five female subjects who were enrolled in the CRC screening program also accepted the invitation for BC screening by MMG. Fourteen subjects eventually refused MMG (0.8%), one is still pending for MMG at the time of writing of this manuscript, and 1 passed away before MMG was offered. Among the 1,719 subjects who underwent MMG, BI-RADS 5 was identified in six (0.3%) and all were confirmed to be breast cancer by biopsy. Among the 37 subjects who had BI-RADS 4 (2.2%), nine had biopsy-confirmed breast cancer, 26 were found to have non-malignant lesions, and two refused biopsy. One hundred forty subjects had BI-RADS 3: 80 were confirmed to be unremarkable after a repeated MMG, while 60 are still waiting for a repeated test. One thousand five hundred thirty-six (89.4%) subjects who had BI-RADS 1 or 2 were advised to repeat MMG in 2 years (**Figure 4**). No complication related to biopsy was reported.

### Defaulted Further Investigations on Positive Screening Tests

In this pilot study, when a subject came forward for CRC screening, the acceptance rates for PC screening or BC screening were 100% and 99.2%, respectively. There were a few who defaulted subsequent investigations due to the invasiveness of the tests, i.e. prostate biopsy or breast biopsy. Five out of 347 (1.4%) subjects who were FIT-positive defaulted colonoscopy; five out of 92 (5.4%) PSA-positive males that who required a prostate biopsy defaulted the procedure; and two out of 43 (4.7%) MMG-positive females requiring ultrasound-guided breast biopsy refused the investigation.

## Discussion

In this feasibility study of a multiple cancer screening program in Hong Kong we observed that if an individual has accepted colorectal cancer screening with FIT, the chance of him accepting an additional screening test for prostate cancer (using PSA) or her accepting an additional screening test for breast cancer (undergoing MMG) is very high (over 99%).

The population in this pilot study is representative of the general population in Hong Kong. They came from all four regions of Hong Kong namely HK Island (13.5%), Kowloon (26.8%), New Territories (58.5%), and Islands (1.3%), compatible with the population distribution of the territory. The education level is also compatible with the overall educational pattern of Hong Kong. There was a fairly even distribution of income among those who participated in the program, probably a result of the free services provided in this program, but also indicated that it is not the under-privileged group who will take advantage of this program. Self-assessment of cancer risk seems to be an important consideration as family history of cancer, obesity and diabetes, smoking and drinking are among the groups with higher participation rates. Family history seems to be an important predictor of participation as 372 (12.5%) subjects had first degree relatives (FDR) with CRC, 170 (9.8%) subjects had FDR with BC, and 49 (3.6%) subjects had FDR with PC.

This one-stop approach of cancer screening was pioneered in the integrated cancer prevention program from Israel. When a battery of tests for 11 cancers were offered to asymptomatic individuals, the compliance rates for mammography and colonoscopy were reported to be significantly higher. The authors reported finding more cancers in this integrated approach than performing cancer screening on individual cancers in the general population. Advanced age, family history of cancer and certain lifestyle parameters were found to be associated with increased cancer risk and the convenience of one-stop investigation was considered to be important for the success. However, evidence of screening for endometrial cancer, skin cancer, cancer of oral cavity, testicle and thyroid were lacking and a screening program for such a long list of malignant diseases is not cost-effective (15).

In a systematic review, seven reports were found summarizing measures of screening for three cancers among females, namely cervical, breast and colorectal cancers (12, 17–21). The results of adherence to three cancer screening ranged from over 50% to only 8% in these studies. Overall, interventions were successful in increasing adherence to multiple screening except for one study with a small sample size (21). Data on adherence to multiple cancer screening are few, and the authors pointed out that an integrated cancer screening measures can provide additional insights into the needs to target population that can help craft strategies to improve uptake and adherence to cancer screening program.

In our study, when a subject come forward for CRC screening, they were given more information about prostate and breast cancer risk. They were also introduced to the options of also screening for BC (the most common female cancer) and PC (the second most common male cancer) at the same setting. A very high acceptance rate for a second cancer screening test is found. This is an encouraging result as launching three separate cancer screening program and inviting individuals (males or females) to undergo separate tests in separate site on separate occasions are likely to have lower uptake and adherence rate. It is possible that this is a self-selected group of health-conscience individuals who are more willing to accept a second cancer screening when they come forward for FIT. In addition, the convenience of screening program, the provision of knowledge and counseling for individual cancers, and the free-of-charge services are the three most important factors for high acceptance of tests in this program. Default to subsequent follow up, by colonoscopy, prostate biopsy, or breast biopsy, occurs but the numbers are relatively small. Despite these defaulted cases, there are quite a substantial number of malignant (cancers of the prostate, breast, and colon/rectum) as well as premalignant lesions (advanced adenoma) diagnosed.

This study is meant for testing the feasibility of having three cancer screening programs combined in one to provide a one-stop service. Our experience has shown that the logistics are feasible but need to be tested in a much larger scale if it is to be taken as a healthcare policy. The cost-effectiveness of this one-stop cancer screening approach remains unknown, as this study was designed to assess its feasibility and effectiveness, but not the cost-effectiveness. This feasibility study is limited by the relatively small number of cases. The final effectiveness, and the identification of factors leading to success of such program will be unveiled in a larger population when the enrollment of 10,000 is accomplished. Secondly, in this protocol, there is a hierarchy of first introducing CRC screening, and then PC screening for males and BC screening for females. There is a lack of information for those who come forward for PC screening and being asked for participating in CRC screening, or those who come forward for BC screening and being asked to join the BC screening. However, we consider this a pragmatic approach as CRC screening is most well received in the public and FIT test are totally non-invasive. Using CRC screening as a first step and introducing a second cancer screening in the same setting provide convenience and lower hurdles for participations. Thirdly, this study may be criticized for creating a high adherence rate merely because all services are offered for free. As we have pointed out, there is no trend of higher acceptance in the low-income population and the average education levels of the participate may dispel some of this skeptic. On the other hand, providing screening for the three most common cancers by the health authority of regional government should certainly eliminate the problem of inequality of healthcare, a target for all government and policy makers to consider.

In conclusion, our pilot study shows that offering CRC, PC and BC screening in the same clinic setting is feasible and may attract more asymptomatic subjects that fulfill screening criteria to participate. As uptake and adherence of cancer screening are major obstacles in cancer prevention program, more studies should be conducted to test various ways of integrating cancer screening program in different jurisdictions.

## Data Availability Statement

The raw data supporting the conclusions of this article will be made available by the authors, without undue reservation.

## Ethics Statement

The studies involving human participants were reviewed and approved by Joint Chinese University of Hong Kong – New Territories East Cluster Clinical Research Ethics Committee. The patients/participants provided their written informed consent to participate in this study.

## Author Contributions

Conceptualization, investigation, methodology by JJYS, SYSW, EYWC, SSMN, ACFN, WCWC, PKFC and PSYC; data curation and analysis by AKCL and TYTL; funding acquisition by JJYS; writing - original draft by JJYS and ACKL; writing - review and editing by SYSW, EYYC, SSMN, ACFN, PKFC, SHW, TYTL, ACKL and JJYS; and supervision by JJYS.

## Funding

This multi-cancer screening program is fully funded by the Hong Kong Jockey Club Charities Trust.

## Conflict of Interest

The authors declare that the research was conducted in the absence of any commercial or financial relationships that could be construed as a potential conflict of interest.
